# Role of Protein Kinase A-Mediated Phosphorylation in CFTR Channel Activity Regulation

**DOI:** 10.3389/fphys.2021.690247

**Published:** 2021-06-11

**Authors:** Angela Della Sala, Giulia Prono, Emilio Hirsch, Alessandra Ghigo

**Affiliations:** ^1^Molecular Biotechnology Center, Department of Molecular Biotechnology and Health Sciences, University of Turin, Turin, Italy; ^2^Kither Biotech S.r.l, Turin, Italy

**Keywords:** cystic fibrosis transmembrane conductance regulator, protein kinase A, phosphorylation, cystic fibrosis, VX770, VX809, F508del-CFTR mutation

## Abstract

Cystic fibrosis transmembrane conductance regulator (CFTR) is an anion channel expressed on the apical membrane of epithelial cells, where it plays a pivotal role in chloride transport and overall tissue homeostasis. CFTR constitutes a unique member of the ATP-binding cassette transporter superfamily, due to its distinctive cytosolic regulatory (R) domain carrying multiple phosphorylation sites that allow the tight regulation of channel activity and gating. Mutations in the *CFTR* gene cause cystic fibrosis, the most common lethal autosomal genetic disease in the Caucasian population. In recent years, major efforts have led to the development of CFTR modulators, small molecules targeting the underlying genetic defect of CF and ultimately rescuing the function of the mutant channel. Recent evidence has highlighted that this class of drugs could also impact on the phosphorylation of the R domain of the channel by protein kinase A (PKA), a key regulatory mechanism that is altered in various CFTR mutants. Therefore, the aim of this review is to summarize the current knowledge on the regulation of the CFTR by PKA-mediated phosphorylation and to provide insights into the different factors that modulate this essential CFTR modification. Finally, the discussion will focus on the impact of CF mutations on PKA-mediated CFTR regulation, as well as on how small molecule CFTR regulators and PKA interact to rescue dysfunctional channels.

## Introduction

The cystic fibrosis transmembrane conductance regulator (CFTR) is a cAMP-activated chloride and bicarbonate channel expressed at the apical surface of secretory epithelia, including the airways, sweat glands, gastrointestinal tract, and other tissues (Riordan et al., 1989; Rommens et al., 1989; Riordan, 2008). The CFTR has a critical role in transepithelial ion and fluid secretion and homeostasis, and mutations in this gene have been implied in the pathogenesis of cystic fibrosis (CF; Li and Naren, 2005). CF, the most common life-shortening rare disease among Caucasians, is an autosomal recessive genetic disease affecting around 32,000 individuals in Europe and about 85,000 individuals worldwide (Zolin et al., 2020). The absence of a functional CFTR leads to a decrease in chloride ion secretion that, together with the consequent alteration of water homeostasis, results in the accumulation of dehydrated mucus, recurrent bacterial infection, and ultimately organ failure. Although CFTR mutations cause a multiorgan disease, respiratory failure is the major cause of morbidity and mortality for CF patients (Ratjen et al., 2015). Although CF nowadays remains an incurable disease, the identification of the defective *CFTR gene* in 1989 (Riordan et al., 1989; Rommens et al., 1989) has prompted significant advances in the development of molecular therapies aimed at addressing the underlying cellular defect (De Boeck and Amaral, 2016; Veit et al., 2016).

The *CFTR* gene, expressed on the long arm of chromosome 7 (Kerem et al., 1989), encodes for a unique member of the large protein superfamily of ATP-binding cassette (ABC) transporters (Klein et al., 1999), which carries a cytosolic regulatory domain that is actively phosphorylated (Collins, 1992; Serohijos et al., 2008). Whereas most of the ABC transporters are active pumps using ATP as the energy source for the transport of small molecules, CFTR is an ATP-gated ion channel where ATP hydrolysis controls channel opening (Dean et al., 2001). Cystic fibrosis transmembrane conductance regulator, like other ABC transporters, is composed of two nucleotide-binding domains (NBDs), involved in channel regulation through ATP binding and hydrolysis, and two transmembrane domains (TMDs), containing six helices that span the plasma membrane to form the ion channel pore (Riordan et al., 1989; Callebaut et al., 2018). In the CFTR, however, the NDB1 domain is linked to the NDB2 one by an additional structural region, a distinctive cytoplasmic regulatory (R) domain with many charged residues and multiple phosphorylation sites that allow the tight regulation of channel activity and gating.

**Figure 1:**
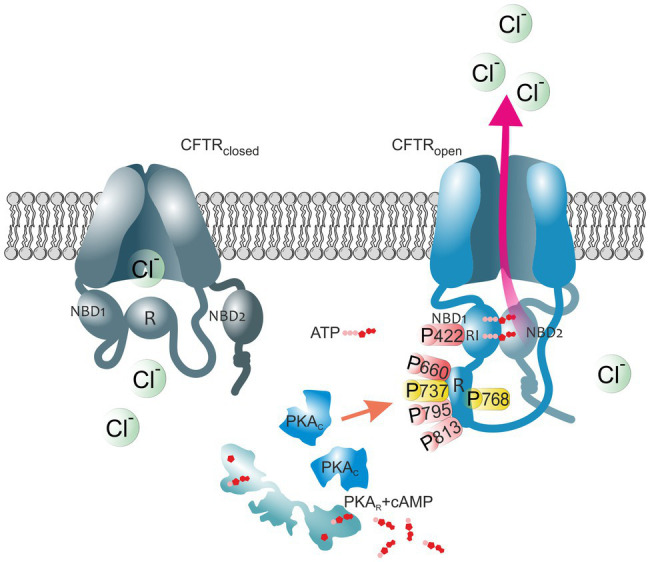
The impact of PKA-mediated phosphorylation on CFTR channel. The cystic fibrosis transmembrane conductance regulator (CFTR) structure consists of two membrane spanning domains, two nucleotide-binding domains (NBD1 and NBD2), and the unique cytoplasmic regulatory (R) domain. Among these, the latter represents a critical site of channel regulation due to its enrichment in protein kinase A (PKA) consensus motifs, with multiple serines and threonines as phosphorylation targets. Moreover, there is an additional phosphorylation site in the regulatory insertion (RI) segment of NBD1. In the closed CFTR state (left channel), the NBD1 interacts with the R domain creating steric hindrances which prevent it from dimerization with NBD2. Upon PKA-dependent phosphorylation (right channel), large conformational changes occur that decrease this interaction leading to the release of the R region from its inhibitory position and allow NBDs dimerization, ultimately ATP binding and CFTR-mediated chloride secretion. Thus, the open probability of the channel is dependent on the access of PKA to the main phospho-sites (S422, S660, S737, S768, S795, and S813). Phospho-sites critical for CFTR channel activation are shown in red. Phosphoserines S737 and S768 are shown in yellow since they have been shown to either activate or inhibit CFTR gating. The role of the major PKA consensus sites in the regulation of CFTR function is detailed in Table 1.

Importantly, the gating of the channel is strictly coupled to the phosphorylation of the R domain by protein kinase A (PKA; Egan et al., 1992; Vergani et al., 2005a,b) and protein kinase C (Chappe et al., 2003; Seavilleklein et al., 2008). PKA-dependent phosphorylation triggers large conformational changes that remove the R region from its position and allow NBDs dimerization to occur. Consequently, ATP binds to the CFTR leading to the opening and activation of the channel, while ATP hydrolysis closes it (Gunderson and Kopito, 1995; Figure 1). In addition to regulating the gating of the channel, PKA-mediated phosphorylation is involved in the regulation of multiple processes, such as CFTR trafficking and stability at the plasma membrane (Chin et al., 2017a).

This review will discuss the role of PKA in CFTR activity regulation, and how this physiological mechanism of channel activation is disrupted by CF mutations. Furthermore, in light of the recent advances in the development of CFTR modulators, we will discuss the impact of these novel molecules on the PKA-mediated regulation of mutant channels.

## CFTR Gating Requires PKA-Mediated Phosphorylation

Although the molecular mechanisms underlying CFTR activation have been studied for years, several questions remain unanswered. This section discusses how channel gating strictly depends on the intrinsic structure of the CFTR protein and several posttranslational modifications, primarily phosphorylation.

One of the major challenges to the understanding of the regulation by the R region of the opening and the gating of CFTR has been the resolution of the CFTR structure. A crucial breakthrough has been represented by the construction of medium-high-resolution models of the 3D structures of the full-length CFTR, first from zebrafish (Zhang and Chen, 2016) and then from humans (Liu et al., 2017). These molecular structures of the CFTR, determined using cryo-electron microscopy, provide insights into a dephosphorylated, ATP-free conformation, which represents a closed and inactive state of the channel (Zhang and Chen, 2016; Liu et al., 2017). Of note, Liu and colleagues with the resolution of the human CFTR structure reveal a previously unresolved helix belonging to the R region docked in an inward-facing conformation between the two halves of CFTR, where it acts as a steric block precluding channel opening (Liu et al., 2017). On the other hand, activation of the CFTR channel is strictly coupled to the formation of a closed NBD dimer, and thereby to the initial conformational changes driven by the PKA-dependent phosphorylation of the R domain (Liu et al., 2017; Mihalyi et al., 2020).

Thus, the open probability of the channel is dependent on the access of PKA to the multiple consensus sites in the R domain (Liu et al., 2017), including 18 potential phosphorylation sites (12 serines and 8 threonines) on a 200 residues long domain. Specifically, in the fully phosphorylated protein, eight phosphoserines (residue positions 660, 700, 712, 737, 753, 768, 795, and 813) and partial phosphorylation of serine at position 670 (Baker et al., 2007) have been detected by mass spectrometry (Townsend et al., 1996; Neville et al., 1997) and NMR (Baker et al., 2007). Recently, Lucaks and colleagues implemented a novel CFTR affinity enrichment method, followed by an advanced mass spectrometry technique, to establish the phospho-occupancy of CFTR upon PKA phosphorylation (Schnur et al., 2019). Among the previously described 15 PKA consensus sites, they identified 10 PKA phospho-sites in the R and NBD1 domain of CFTR (serine 422, 660, 670, 686, 700, 712, 737, 753, 768, and 795; Schnur et al., 2019). Table 1 provides a comprehensive description of the major phospho-sites and their effect on CFTR channel activity regulation. The role of each phosphoserine in the modulation of CFTR activity is quite complex and multiple experiments have been attempted to address this problem by site-direct mutagenesis, where the putative phosphorylation sites in the R region were substituted with alanines (Kanelis et al., 2010; Marasini et al., 2012, 2013). Of note, among such studies, the replacement of serine at position 700, 795, and 813 revealed a decrease in channel open probability (Rich et al., 1993; Vais et al., 2004; Chen, 2020), whereas mutations of serine 737 and 768 increased the activity, thus implying a phospho-dependent inhibitory effect of these residues on the CFTR channel function (Wilkinson et al., 1997; Kongsuphol et al., 2009). Accordingly, phosphorylation of these inhibitory sites by AMPK inhibits CFTR-mediated chloride secretion by maintaining the channel in a closed state (Kongsuphol et al., 2009). These findings appear to be in apparent contrast with the results of the work by Riordan and colleagues aimed at investigating whether individual PKA phospho-sites act cooperatively or distinctly in the regulation of channel activity. This study investigated the impact of the reintroduction of S737 and S768 in a CFTR variant insensitive to PKA, namely, the 15SA mutant, in which the 15 PKA consensus sites were replaced by alanines (Hegedus et al., 2009). Reintroduction of S737 or S768 or both restored a significant level of channel activation by PKA, thus indicating that phosphorylation of these sites stimulates CFTR channel activity. Furthermore, a mutant CFTR in which all phosphorylation sites have been removed, completely eliminates the PKA-dependent regulation of the channel activity (Xie et al., 2002). In line with this finding, a CFTR mutant containing six or more serine-to-aspartate substitutions mimicking the effect of phosphorylation results in channel opening even in the absence of PKA (Rich et al., 1993).

**Table 1:** PKA phospho-sites in CFTR and their role in channel activity regulation.

PKA consensus site	Domain	Mechanisms involved in PKA-mediated phosphorylation	References
Serine-422	RI-domain of NBD1	Phosphorylation disrupts the interaction with S660 in the R domain promoting NBDs dimerization, formation of the two ATP-binding pockets and CFTR activation	Hudson et al., 2012; Dawson et al., 2013
Serine-737, 768	R-domain	Phosphorylation leads to CFTR channel opening in the 15SA CFTR variant insensitive to PKA. In contrast, phosphorylation by AMPK of these sites maintains CFTR in a closed state	Hegedus et al., 2009; Kongsuphol et al., 2009
Serine-768, 795, 813	R-domain	Phosphorylation leads to CFTR binding to the 14-3-3b isoform in the ER and promotes its forward trafficking to the cell surface	Liang et al., 2012
Serine-660, 700, 795, 813	R-domain	Phosphorylation leads to conformational change and release of the R domain from its inhibitory position, promoting NBDs dimerization and CFTR gating	Rich et al., 1993; Vais et al., 2004; Chen, 2020

Overall, these experiments highlight that the effects of PKA-dependent phosphorylation of the R domain are correlated to conformational changes triggered by the negative charges of the phosphate group introduced on phosphoserines (Zhang et al., 2017, 2018; Schnur et al., 2019).

As a confirmation, circular dichroism, X-ray scattering, and NMR experiments showed a reduced density of the phosphorylated structure, suggesting that upon phosphorylation the R domain becomes entirely disordered and less compact (Baker et al., 2007; Bozoky et al., 2013). Further evidence of the structure of phosphorylated CFTR, in its ATP-bound conformation, comes from cryo-electron microscopy (Zhang et al., 2017, 2018). These images, comparing the conformation in a phosphorylated and ATP-bound state (Zhang and Chen, 2016; Liu et al., 2017) with the dephosphorylated and ATP-free conformation (Zhang et al., 2017, 2018), define the structural changes of the two human and zebrafish CFTR orthologs. These structures further reveal the clearly distinct position of the R domain after phosphorylation. Upon PKA binding, phosphorylation promotes the release of the R domain from its inhibitory position, causing NBDs dimerization and flipping of the TMDs into an outward-facing conformation, ultimately leading to channel opening and activation of its ATPase function (Sorum et al., 2015; Figure 1).

On the other hand, how phosphorylation triggers the ATPase activity of CFTR has remained unclear. Whereas PKA-mediated phosphorylation of the R domain is absolutely required (Chang et al., 1993; Winter and Welsh, 1997; Seibert et al., 1999), whether ATP binding is essential for CFTR gating is still controversial. In the following paragraph, we will describe how phosphorylated CFTR channels are gated through ATP-dependent and independent mechanisms.

## Effects of PKA-Mediated Phosphorylation on ATP Binding

The widely accepted model of CFTR activation establishes that phosphorylated CFTR channels are gated through an ATP-dependent mechanism that is based on conformational changes (Sorum et al., 2015). In these models, the ATP molecule powers the gating cycle by inducing the opening through NBD1:NBD2 dimerization as well as the closing through its hydrolysis (Higgins and Linton, 2004). While PKA phosphorylation promotes the release of the R domain from its position, the binding of ATP triggers the dimerization of the NBDs that, in turn, leads to channel opening and activation (Vergani et al., 2003, 2005b; Csanady et al., 2010). Therefore, the phosphorylation of the R domain increases the rate of channel opening by stimulating conformational changes that enhance the affinity for ATP (Winter and Welsh, 1997).

Following this obligatory modification, the ATP generally bound only to NBD1 is released, and it binds to the two sites at the NBD1:NBD2 interface (Aleksandrov et al., 2018). Recent studies by Csana and colleagues support a model wherein ATP binding to both NBDs enhances their dimerization and consequently channel opening (Mihalyi et al., 2016). Instead, the ATPase activity at NBD2 subsequently promotes destabilization of the NBD dimer, leading to channel closure (Mihalyi et al., 2016). Additionally, Lewis and colleagues have found that in the CFTR closed state, the NBDs dimer is prevented, due to the inhibitory interaction of the R domain with the regulatory insertion (RI) domain of NDB1 (Lewis et al., 2004). Although the majority of the canonical phosphorylation sites are located within the R domain of CFTR, an additional residue (S422) in the RI region plays a key regulatory function (Lewis et al., 2004). In particular, S422 of the RI domain interacts with S660 in the R domain, creating steric hindrances on NBD1, which prevent it from dimerization with NBD2 (Hudson et al., 2012; Dawson et al., 2013). Upon phosphorylation by PKA of these two serines, the alpha-helical content of both the RI and the R regions decreases (Hudson et al., 2012; Dawson et al., 2013). Therefore, phosphorylation by PKA weakens the interaction between the R domain and NBD1, ultimately allowing the formation of the NBD1:NBD2 dimer (Aleksandrov et al., 2018).

Different from the wild-type CFTR channel, where the gating is dependent on ATP binding, in gating mutants where ATP binding is impaired, the introduction of a second mutation, like the K978C or the NBD2 deletion, restores the open conformation of the channel, but PKA-mediated phosphorylation of the R domain is still required for full channel activation (Eckford et al., 2012). These examples of ATP-independent CFTR channel appear to be regulated by phosphorylation, *via* allosteric interactions between the R domain and the NBD1 region (Eckford et al., 2012). Nonetheless, further studies are required to understand the molecular basis for such ATP-independent gating and CFTR regulation.

Overall, phosphorylation represents an additional level of control of CFTR activity besides ATP binding and hydrolysis. Given that the ATP concentrations within a cell are high enough for constant activation of the channel, phosphorylation is essential for the prevention of futile cycling of the channel between the open and closed conformations (Higgins and Linton, 2004).

## Effects of CF-Causing Mutations on PKA-Mediated Phosphorylation of CFTR

Considering the essential role of PKA-dependent phosphorylation of the intrinsically disordered R domain in modulating CFTR gating (Hallows et al., 2003; Billet et al., 2015, 2016), it is plausible that CF-causing mutations may disturb this mechanism of channel regulation. The most common CFTR mutation, consisting of the deletion of phenylalanine (F508del) located at the interface between NBD1 and the intracellular loop 4, interferes with the correct R domain folding and assembly (Riordan, 2005; Hwang et al., 2018). As a consequence, the aberrant channel is targeted to early ER-associated degradation (Farinha and Amaral, 2005). Moreover, the resulting thermal instability of this mutant form further contributes to its removal from the plasma membrane (Okiyoneda et al., 2010), which has been shown to contain only 2% of mature F508del-CFTR compared to the normal wild-type (WT) channel amount (Van Goor et al., 2011). Importantly, Wang and colleagues demonstrated that the phosphorylation rate of F508del-CFTR by PKA is significantly lower than that of WT-CFTR, suggesting that the abnormal protein does not constitute a good substrate for the kinase (Wang et al., 2000). The same results were obtained from the analysis of the other two disease-causing CFTR mutations, R170G and A1067T, both occurring in coupling helices that allow the correct channel activation by phosphorylation (Chin et al., 2017b).

Furthermore, a relevant study by Pasyk et al. exploited mass spectrometry to quantitatively assess PKA-mediated phosphorylation of serine 660 of F508del-CFTR at the ER and reported a drastic reduction of phosphorylation upon forskolin stimulation in comparison with the WT form (Pasyk et al., 2009). Interestingly, COPI-mediated retrograde trafficking from the Golgi to the ER, which prevents misfolded F508del-CFTR from successfully reaching the apical cell membrane (Rennolds et al., 2008; Okiyoneda et al., 2010), has been linked to phosphorylation-dependent interactions between the channel and 14-3-3 proteins (Liang et al., 2012). In particular, selective binding of 14-3-3 protein isoforms to specific PKA-phosphorylated sites within the R region decreases this retrograde retrieval, ultimately resulting in augmented CFTR biogenesis. Unfortunately, whether the impaired phosphorylation caused by the deletion of F508 negatively impacts this protein-protein interaction has yet to be elucidated.

Another relevant process that contributes to the maintenance of channel density at the plasma membrane is the endosomal trafficking of the CFTR, which ensures the internalization and recycling of abundant or misfolded proteins (Moore et al., 2007). Notably, Holleran and collaborators revealed that cell surface F508del-CFTR is targeted to lysosomes and displays defective PKA-regulated exocytosis, consisting of significantly slower rates of translocation to the plasma membrane from the Rab11+/EHD1+ endosomal recycling compartment upon PKA challenge (Holleran et al., 2013). Therefore, these data suggest that the abnormal phosphorylation of the mutant CFTR by PKA could, at least partly, account for the defective peripheral trafficking of the channel. Moreover, it has been shown that the excision of the RI polypeptide from the NBD1 of F508del protein, which contains the additional PKA consensus site S422, may be beneficial for the life cycle of the mutant channel (Aleksandrov et al., 2010). Specifically, removal of this region was found to promote the escape of F508del-CFTR from the ER quality control machinery and thus the increased apical surface stability. In addition, recent evidence confirmed that the amount of phosphorylated S422, which represents a minor PKA target site, is decreased in the aberrant F508del protein (Pankow et al., 2019). However, further analyses are required to clarify whether the presence of the RI hampers the F508del-CFTR stability independently of its phosphorylation state and to evaluate the biological consequence of its reduced phosphorylation in the mutant CFTR.

Another important CFTR genetic defect that could potentially interfere with PKA-mediated phosphorylation of the channel is the G551D mutation, a class III mutation with a worldwide frequency of ∼4% (CFF patient registry, 2019). The disease-causing substitution occurs in the NDB1 domain and strongly impairs the ATP-dependent channel gating, without hindering channel trafficking to the plasma membrane (Gregory et al., 1991; Bompadre et al., 2007). Interestingly, Chang and colleagues demonstrated that the R domain of this mutant form undergoes normal phosphorylation despite its lack of ATP binding (Chang et al., 1993). In contrast, a very recent study showed that G551D-CFTR exhibits defective phosphorylation-dependent activation as a result of a decreased sensitivity to PKA-mediated phosphorylation. Stimulation of mutant channels with high doses of PKA induced a remarkable increase in their activity, thus suggesting that the intrinsic phosphorylation defect of G551D-CFTR might be one of the major causes of low basal functionality. Moreover, phosphorylation of the serine residue 737 of G551D occurred at a lesser extent compared to WT-CFTR, highlighting a possible causative factor of the slower activation rate of the aberrant protein (Wang et al., 2020).

Taken together, abnormal PKA-mediated phosphorylation underlies multiple molecular defects observed in mutant CFTR channels and represents a promising therapeutic target for the treatment of CF. Therefore, the following paragraph will focus on how currently available CFTR modulators impact on the phosphorylation of the channel by PKA.

## Effects of CFTR Modulators on PKA-Dependent Regulation of CFTR

In recent years, major advances have been made in the field of precision therapy against the underlying cause of CF. A variety of small molecules, designed to target specific channel defects and collectively known as CFTR modulators, have been developed to improve or even restore the expression, activity, and stability of defective CFTR variants (Lopes-Pacheco, 2016). Of outmost relevance are CFTR correctors, which rescue the misprocessing of mutant CFTRs, and potentiators, that are intended to restore the defective cAMP-dependent chloride channel activity at the cell surface (Lopes-Pacheco, 2016). Nonetheless, how these therapeutic agents act on the PKA-mediated phosphorylation of CFTR mutants is still largely unaddressed.

Importantly, there are some paradigmatic exceptions of CFTR modulating drugs whose relationship with PKA-dependent phosphorylation has been investigated and will be now discussed. The potentiator VX-770 (ivacaftor) is approved both as a single therapy for G551D mutants and as a combination with either the corrector VX-809 (lumacaftor; Connett, 2019) or VX-445 and VX-661 for F508del carriers (Bear, 2020). Importantly, ivacaftor was found to enhance channel activity in an ATP-independent manner (Eckford et al., 2012). However, non-phosphorylated CFTR does not exhibit any significant ion current increase upon VX-770 stimulation, suggesting that its potentiating action is strictly dependent on the phosphorylation state of the channel (Eckford et al., 2012). Conversely, Jih and collaborators demonstrated that this potentiator can increase the activity of a CFTR lacking the R domain, thus arguing with the previous hypotheses of a phosphorylation-dependent mechanism (Jih and Hwang, 2013). Recently, a study by Uliyakina et al. revealed that the absence of the RI domain strongly emphasizes VX-809-mediated rescue of F508del-CFTR but negatively impacts the channel currents stimulated by VX-770 (Uliyakina et al., 2020). Therefore, these two modulators display a contradictory behavior in the absence of the unique region containing the PKA phosphorylation site, S422, but future analyses are needed to dissect the role of RI in mutant CFTR. Additionally, another molecule, VRT-532, which had been formerly identified as a potentiator (Van Goor et al., 2006), showed to significantly amplify the activity of G551D-CFTR mutants. Despite its direct interaction with the aberrant channel (Pasyk et al., 2009), VRT-532 did not induce an increase in CFTR phosphorylation, suggesting that its mechanism of action occurs at stages that are downstream of the PKA kinase activity (Pyle et al., 2011).

Conversely, correctors based on 3-(2-benzyloxy-phenyl)-5-chloromethyl-isoxazoles, like UCCF-152, were found to stimulate potent and rapid phosphorylation of the R domain of WT, temperature-rescued F508del-CFTR and G551D-CFTR, while also increasing iodide currents, leading to their classification as CFTR activators (Pyle et al., 2011). The specific residues phosphorylated upon the interaction with UCCF-152 have not been yet identified, but this isoxazole neither raises cellular cAMP levels nor directly activates PKA, suggesting a possible enhancement of the propensity of the R domain to be phosphorylated by releasing steric hindrances (Sammelson et al., 2003). To date, no additional analyses have been performed to further characterize UCCF-152 as a candidate CF-treating agent. The impact of CF-causing mutations on PKA phosphorylation of CFTR, and the effect of CFTR modulators on the activity of mutant channels is summarized in Table 2.

**Table 2:** Effects of CFTR modulators on PKA-dependent regulation of CFTR mutants.

CFTR mutant	Impact on PKA-mediated phosphorylation	Effects of CFTR modulators	References
F508del	Reduced phosphorylation rate (PKA phospho-sites S442, S660), altered interaction with 14-3-3 proteins, increased COPI-mediated retrograde trafficking, defective PKA-regulated exocytosis	Potentiation by VX-770 shows contrasting results on phosphorylated and non-phosphorylated mutant CFTR. VX-770-mediated rescue requires S442, while its deletion enhances VX-809 correction. The activator UCCF-152 potentiates the phosphorylation of the R domain	Wang et al., 2000; Pasyk et al., 2009; Pyle et al., 2011; Eckford et al., 2012; Liang et al., 2012; Holleran et al., 2013; Uliyakina et al., 2020
G551D	Reduced phosphorylation (PKA phospho-site S737) and defective phosphorylation-dependent activation	VRT-532 potentiates the activity of the channel. The activator UCCF-152 potentiates the phosphorylation of the R domain	Chang et al., 1993; Pyle et al., 2011; Wang et al., 2020

Overall, our current knowledge of how CFTR modulators interfere or promote PKA-dependent phosphorylation of the channel remains scarce, and future efforts are needed to allow a better understanding of their impact on this essential molecular modification of the channel.

## Conclusion

In conclusion, PKA-dependent phosphorylation plays a key role in multiple steps during the life cycle of CFTR. While interactions resulting in phosphorylation at the CFTR R domain regulate channel opening and activity, other phosphorylation events at the C and N terminal ends of CFTR modulate channel stability and trafficking at the PM. Importantly, CFTR mutations leading to CF impair different steps of CFTR biogenesis that are regulated by these phosphorylation events. Targeting PKA-mediated phosphorylation thus represents a promising strategy to rescue the activity of different CF-causing CFTR variants. Nonetheless, how correctors and potentiators, including their highly effective combinations, like the recently approved Trikafta, impact on the PKA-mediated phosphorylation of CFTR still needs to be thoroughly investigated. Future studies in this direction might help to maximize therapeutic efficacy, ultimately normalizing the life expectancy of CF patients.

## Author Contributions

ADS designed the overall layout and wrote the bulk of the manuscript. GP was involved in the writing of the manuscript and referencing. EH reviewed the manuscript and produced the figure. AG was involved in the editing and critical revision of the manuscript. AG and EH acquired the funding. All authors contributed to the article and approved the submitted version.

### Conflict of Interest

AG and EH are founders and board members of Kither Biotech, a company focused on the development of PI3K inhibitors for airway diseases not in conflict with statements in this article. The other co-authors declare no conflict of interest.
